# Room-Temperature Carbon Dioxide Gas Sensor Based on Co-Ferrite Nanoparticles

**DOI:** 10.1021/acsomega.5c11563

**Published:** 2026-02-18

**Authors:** Yogesh Mahor, Dorota Koziej, Cecilia A. Zito

**Affiliations:** † Center for Hybrid Nanostructures (CHyN), Institute of Nanostructure and Solid State Physics, 14915 University of Hamburg, Luruper Chaussee 149, 22761 Hamburg, Germany; ‡ The Hamburg Center for Ultrafast Imaging, 22761 Hamburg, Germany

## Abstract

The development of carbon dioxide (CO_2_) sensors is essential for both environmental and indoor air monitoring applications. Here, we investigate the CO_2_-sensing performance of cobalt ferrite (CoFe_2_O_4_) nanoparticles (NPs) at room temperature under relative humidity (RH) conditions. The NPs exhibit a phase-pure spinel CoFe_2_O_4_ structure with an average particle size of approximately 6 nm. The fabricated sensors demonstrate high CO_2_ sensitivity, showing a nearly linear response over concentrations ranging from 250 to 4000 ppm, with stable reproducibility over repeated exposure cycles and prolonged operation at 30% RH. The sensor demonstrates a broad and practically relevant concentration detection range, exhibiting high sensor signals and reliable response/recovery for real-life applications. Interestingly, higher humidity levels (50% RH) alter the sensing mechanism due to the introduction of parallel conduction pathways promoted by increased water coverage on the surface of the NPs. Furthermore, an increase in crystallite size to 60 nm results in diminished CO_2_ signal, underscoring the critical role of nanosized particles in enhancing gas diffusion and active site availability. This work therefore shows that CoFe_2_O_4_ NPs correspond to a promising material platform for room-temperature CO_2_ detection, with humidity and particle size having a strong influence on their sensing performance.

## Graphical abstract

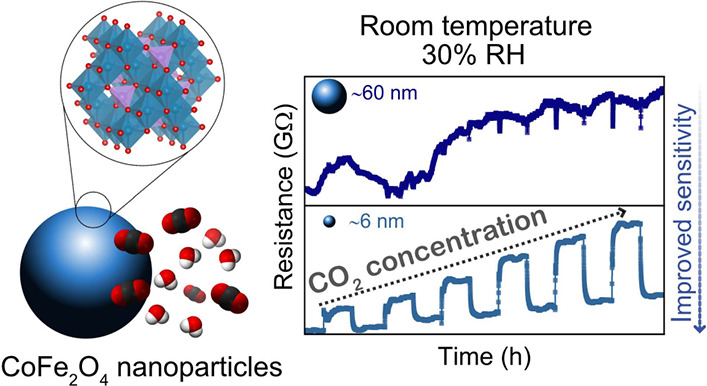

## 1 Introduction

The rising threat of global warming and the increasing levels of atmospheric pollution are becoming serious issues in day-to-day life. The major cause of these environmental problems is the increased amount of greenhouse gases in the atmosphere. Among these gases, carbon dioxide (CO_2_) accounts for more than 70% of greenhouse gas emissions, reaching a new record for its atmospheric level of nearly 423 ppm in 2024, with an annual increase of over 3 ppm.^1^ Furthermore, CO_2_ is hazardous not only to the environment but also to humans, as it is an asphyxiant and an inhalation toxicant. Concentrations above 1000 ppm in indoor environments can cause several health issues, such as drowsiness, discomfort, shortness of breath, cognitive impairments, increased heart rate, and more, which can lead to suffocation and death in confined spaces.^2–4^ Typically, indoor air quality and occupational safety standards define a critical CO_2_ range from approximately 400 ppm (typical atmospheric level) up to 1200 ppm for comfort and productivity, while the EU’s indicative occupational exposure limit value (IOELV) caps an 8 h exposure at 5000 ppm.^5–7^ Hence, the practical working range of a CO_2_ gas sensor should be approximately 400–5000 ppm.

As monitoring air quality to protect human well-being has become essential, new technologies have been developed to overcome the limitations of commercially available state-of-the-art CO_2_ sensors, which are based on nondispersive infrared (NDIR),^8,9^ and electrochemical principles.^10,11^ The most promising approach corresponds to chemiresistive gas sensors, which, unlike the methods employed so far, offer low cost, simple operation, high reliability, and the potential for miniaturization.^12–14^ Numerous metal oxide semiconductor-based materials have been employed as chemiresistive CO_2_ sensors,^15,16^ including CeO_2_, TiO_2_, SnO_2_, SnO_2_/La_2_O_3_, CuO, ZnO, WO_3_, La_2_O_2_Cl_3_, NdO_2_CO_3_, BaTiO_3_–CuO, etc.^17–28^ However, the major drawback for these oxides is the frequent requirement of high operating temperatures (>100 °C).

In recent years, a particular class of metal oxides, spinel ferrites (MFe_2_O_4_), has attracted significant attention due to their excellent chemical stability and surface reactivity, enabling effective gas adsorption. Yet, MFe_2_O_4_-based CO_2_ sensors remain relatively underexplored, with only a few reports on MgFe_2_O_4_, Zr-CaFe_2_O_4_, CuO/CuFe_2_O_4_, SnO_2_/Mn_0.5_Cu_0.5_Fe_2_O_4,_ and NiFe_2_O_4_.^29–36^ Nevertheless, these sensors still require high operating temperatures, which limit their practical applications.

In this study, we report a room temperature (RT) CO_2_ sensor based on cobalt ferrite - CoFe_2_O_4_-nanoparticles (NPs), synthesized via a one-step microwave-assisted solvothermal method. The synthesized NPs, with an average diameter of 6 nm, were evaluated under varying humidity conditions. The sensor exhibited optimal performance at 30% relative humidity (RH), outperforming responses recorded under dry (0% RH) and higher humidity (50% RH) conditions across the CO_2_ concentration range of 250–4500 ppm. The CoFe_2_O_4_-based sensor displays high CO_2_ sensitivity with prolonged stability and reliable reproducibility at 30% RH. Moreover, we show that increasing the particle size to 60 nm resulted in a loss of the sensor signal to CO_2_, elucidating the crucial role of the nanosized particles in enhancing gas diffusion and providing active sites for CO_2_ sensing. These results demonstrate, for the first time, the potential of CoFe_2_O_4_ NPs as a room temperature chemiresistive CO_2_ sensing material for indoor air quality monitoring.

## 2 Experimental Section

### 2.1 Materials

Cobalt­(II) acetylacetonate (Co­(C_5_H_7_O_2_)_2_, >97%) and iron­(III) acetylacetonate (Fe­(C_5_H_7_O_2_)_3_, >99.8%), anhydrous benzyl alcohol (>99.8%), anhydrous ethanol (98%) and ethylene glycol were all purchased from Sigma-Aldrich and used without further purification. Synthetic air (20/80 vol %; O_2_/N_2_) and 1 vol % CO_2_ in synthetic air were purchased from Air Liquide Deutschland GmbH.

### 2.2 Synthesis of CoFe_2_O_4_ NPs

CoFe_2_O_4_ NPs were synthesized through a microwave-assisted nonaqueous sol–gel method adopted from the previously reported protocols.^37–39^ In summary, 1 mmol (353 mg) of Fe­(C_5_H_7_O_2_)_3_ was dissolved in 5 mL of benzyl alcohol in a 20 mL glass vial and completely homogenized. Then, 0.5 mmol (120 mg) of Co­(C_5_H_7_O_2_)_2_ was added to the reaction mixture and stirred for 10–15 min until obtaining a homogeneous mixture. This mixture was then transferred to a 10 mL microwave reaction vial and introduced to the microwave reactor (CEM Discover, 2.45 GHz), where it was heated at 180 °C for 30 min. The color of the mixture turned from blood red to dark brown after the reaction. The NPs were separated from the reaction mixture by centrifugation at 10,000 rpm for 10 min, the supernatant was discarded, and the CoFe_2_O_4_ NPs were washed 5 times with anhydrous ethanol under the same conditions. The precipitate was dried overnight in the oven at 60 °C and stored in powdered form for further characterization.

### 2.3 Characterization Methods

Powder X-ray powder diffraction (PXRD) patterns were measured using a Bruker D8 Advance X-ray diffraction machine equipped with Cu Kα radiation (λ of 1.5418 Å). Scanning/Transmission Electron Microscopy (SEM/STEM) images were collected using a Regulus 8220 microscope (Hitachi High Technologies Corp., Japan), equipped with an X-Max detector (Oxford Instruments, UK) for energy-dispersive X-ray spectroscopy (EDS) measurements. The N_2_ physisorption measurements were conducted at 77 K on a 3Flex gas adsorption analyzer (Micromeritics Instrument Corporation). The specific surface area was calculated using the Brunauer–Emmett–Teller (BET) method. The total pore volume was determined at 0.95 *p*/*p*_0_. The pore size distribution calculations were carried out by using density functional theory (DFT) model considering oxide surface and cylindrical pore geometry. Prior to the measurements, the sample was degassed at 150 °C for 18 h. The water vapor sorption isotherms were measured at 25 °C using VSTAR vapor sorption analyzer (Anton Paar). The sample was degassed at 150 °C for 18 h before the measurements.

Total X-ray scattering and high-resolution PXRD patterns were recorded at P21.1 beamtime at PETRA III at Deutsches Elektronen-Synchrotron (DESY), Hamburg, Germany, using a wavelength of 0.12229 Å. The samples’ powders were loaded into 1.0 mm diameter Kapton capillaries, and the total scattering data were collected using a PerkinElmer XRD1621 detector (2048 × 2048 pixels). Instrumental parameters were obtained from the calibration with LaB_6_. The total scattering patterns were processed into the pair distribution functions (PDF) using xPDFsuite^40^ software with PDFgetX3.^41^ The final PDFs were refined employing PDFgui^42^ with *Q*_damp_ and *Q*_broad_ parameters determined as 0.0185 and 0.0159 Å^–1^, respectively, from the LaB_6_ calibrant.

### 2.4 Sensor Fabrication

20 mg of CoFe_2_O_4_ NPs were taken into a mortar and pestle, and mixed with 10 μL of ethylene glycol to produce a slurry. This slurry was then applied to the alumina substrate with Pt-interdigitated electrodes (IDE; 0.3 mm finger width/spacing) using a thin blade with Kapton tape as spacer. The substrate with sensing film was calcined at 400 °C for 1.5 h at the heating rate of 5 °C/min to remove the ethylene glycol and stabilize the sensing film onto the substrate.

### 2.5 Sensing Performance

The CO_2_ gas sensing measurements were conducted in a continuous flow Teflon chamber at room temperature (20 °C). A computer-controlled gas mixing system, equipped with Bronkhorst mass flow controllers, delivered a constant synthetic air flow of 200 mL/min with tunable relative humidity (RH) and CO_2_ concentration. The RH was controlled by flowing the synthetic air through a water bubbler maintained at 20 °C, which defines the reference temperature for the reported RH values. The sensing film was conditioned in humid air for several hours until a stable baseline was achieved before the sensor was exposed to the first pulse of CO_2_ gas. The changes in the DC resistance of the sensors upon exposure to CO_2_ gas pulses were recorded using a programmable electrometer (Keithley model 617). The sensor signal is defined as the ratio *R*_CO2_/*R*_0_, where *R*_CO2_ is the resistance in the presence of the target gas (CO_2_), and *R*_0_ is the initial resistance in humidified synthetic air without the analyte. For each CO_2_ concentration, the average sensor signal and standard deviation were calculated from multiple recorded exposure cycles. The response time is defined as the time taken to reach 90% of the maximum resistance change upon exposure to the target gas. Similarly, the recovery time is the time needed to reach 90% resistance of the baseline resistance in humidified synthetic air after CO_2_ removal from the chamber for the corresponding pulse.

## 3 Results and Discussion

We investigate the morphology, crystallite size, and composition of the CoFe_2_O_4_ NPs using S­(T)­EM, PXRD, and PDF, as shown in Figure 1. The STEM image in Figure 1a reveals that the sample consists of spherical NPs smaller than 10 nm. Upon deposition of the CoFe_2_O_4_ NPs on the IDE (Figure S1), they form a compact film while retaining their nanoscale dimensions, indicating no significant changes during the sensor fabrication. Additionally, EDS mapping and spectrum confirm the composition of the NPs in the deposited film. Figure S2 shows a homogeneous distribution of Fe, Co, and O throughout the entire sample, with the elemental quantification yielding 26.5 atom % Fe, 12.8 atom % Co, and 57.8 atom % O, indicating the successful synthesis of CoFe_2_O_4_. The phase purity of the CoFe_2_O_4_ NPs is confirmed by high-resolution PXRD analysis, as shown in Figure 1b. All reflections match well with the calculated reference pattern of cubic spinel CoFe_2_O_4_ (space group *Fd3̅m* (227) - ICSD No. 33181).^43^ The crystallite size of the NPs is estimated to be ∼6 nm using the Debye–Scherrer equation applied to the most intense (311) reflection. Moreover, we investigate the effect of calcination at 400 °C for 1.5 h, which is used in the sensor preparation, on the composition and size of the NPs. In Figure S3, the PXRD patterns indicate that the NPs retain the pure spinel CoFe_2_O_4_ phase and the particle size remains unchanged (∼6 nm) after calcination.

**Figure 1:**
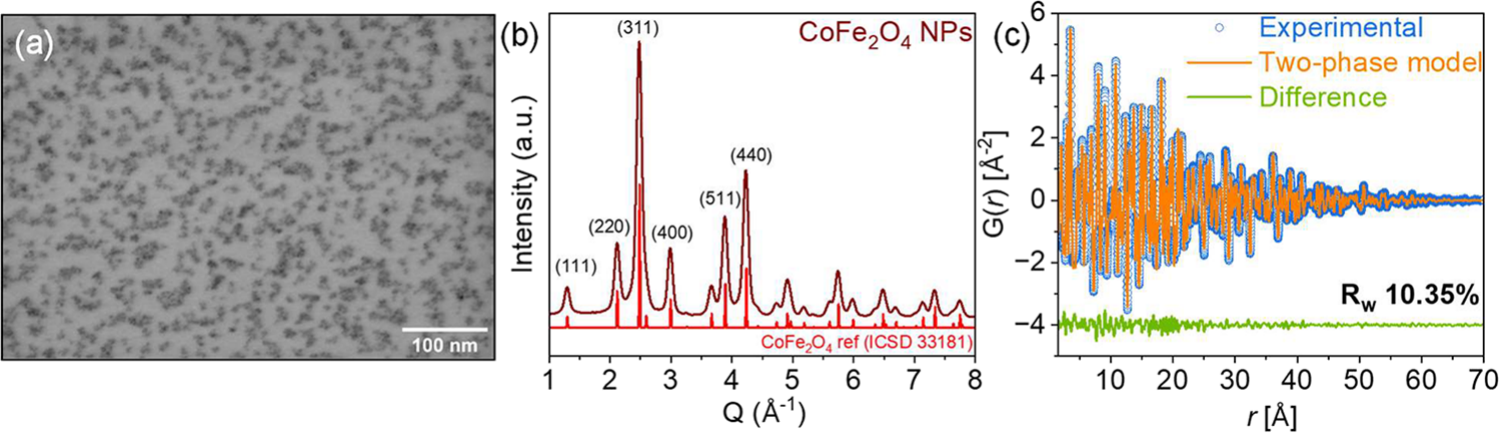
Characterization of the as-synthesized CoFe_2_O_4_ NPs. (a) STEM image. (b) High-resolution PXRD pattern of the CoFe_2_O_4_ NPs. The Bragg peaks for the cubic spinel CoFe_2_O_4_ structure are shown in red lines. (c) Fit between experimental and calculated PDFs (*G*(*r*)) using two identical phases of the cubic spinel structure of CoFe_2_O_4_ with different spd and scale factor parameters.

To further confirm the crystalline size and crystal structure, PDF analysis is employed, as shown in Figure 1c. The experimental PDF was refined using crystallographic data from the cubic spinel structure of CoFe_2_O_4_.^43^ Using a two-phase model, we observe improvements in the fitting compared to single-phase modeling, minimizing systematic deviations in the peak intensities of the PDFs. Modeling the data against two phases of CoFe_2_O_4_, each with distinct spherical particle diameters (spd) and scale factors, yields a good agreement between experimental and calculated data, as reflected in the goodness of the fit (*R*_w_) of 0.103 (see Table S1). The fitting also suggests a size distribution of coherent domains in the range of 29 Å (28%) to 76 Å (72%), consistent with the observed particle size distribution in the STEM images and the crystallite size from PXRD. Therefore, we confirm the phase purity of the as-synthesized CoFe_2_O_4_ NPs and reveal that no significant changes in the phase composition and crystallite size after calcination at 400 °C and sensor fabrication.

We investigate the CO_2_ gas sensing performance of the CoFe_2_O_4_ NPs by monitoring the changes in resistance upon exposure to CO_2_ pulses ranging from 250 to 4000 ppm. We assess sensing performance at room temperature (RT) and 30% relative humidity (RH), as shown in Figure 2. Figure 2a demonstrates the sensor’s resistance changes upon CO_2_ gas exposure, showing a detectable response even at a low concentration of 250 ppm. As the CO_2_ gas concentration increases, the magnitude of the resistance changes also increases, indicating a concentration-dependent change. Figure S4 shows the same behavior for three consecutive cycles of CO_2_ sensing experiments (250–4000 ppm). We observe that the resistance does not return entirely to the initial baseline resistance values between the CO_2_ pulses and a small baseline drift is observed over time. This drift is likely due to slow equilibration of the sensor with ambient air, including oxygen adsorption and humidity. These processes occur slowly at RT.

**Figure 2:**
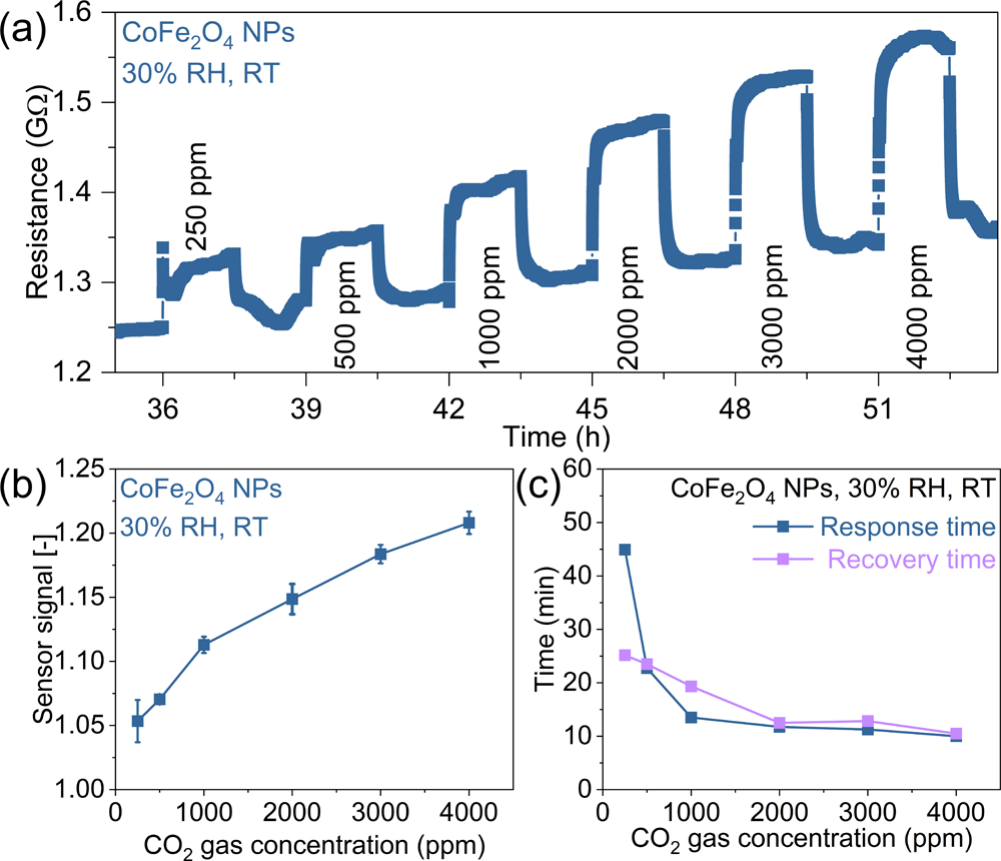
CO_2_ sensing performance of CoFe_2_O_4_ NPs at 30% RH and RT. (a) Resistance changes of the sensor upon exposure to CO_2_ in the concentration range of 250–4000 ppm. (b) Sensor signal as a function of the CO_2_ concentration. (c) Response time and recovery time for the corresponding CO_2_ gas pulses.

We calculate the average sensor signal for each CO_2_ concentration across all three exposure cycles, as given in Figure 2b. The sensor signal increases nearly linearly (Figure S5) with CO_2_ concentration, showing high sensing performance at RT and 30% RH. The average sensor response ranges from 1.05 for the lowest measured CO_2_ concentration (250 ppm) to 1.21 for the highest concentration (4000 ppm). The small standard deviation indicates that the sensor behavior is reliable and reproducible over an extended period and across a wide concentration range, despite the small baseline drift. Figure 2c shows the response and recovery times as a function of the CO_2_ gas concentration, which are essential parameters to be evaluated. The response time decreases with increasing concentration, ranging from ∼45 min at 250 ppm to ∼10 min at the highest CO_2_ concentration (4000 ppm), possibly due to the higher availability of CO_2_ molecules. The recovery time exhibits a similar trend, with values of ∼25 min for 250 ppm and ∼10 min for 4000 ppm.

The observed CO_2_ sensing behavior can be explained by the surface reactions occurring on CoFe_2_O_4_, as described as follows. When exposed to air, O_2_ molecules adsorb on the surface of the CoFe_2_O_4_, extracting electrons from the conduction band and leading to the formation of ionosorbed oxygen species (e.g., O_2_^–^, O^–^, and O^2–^).^44^ This process results in the formation of a depletion layer near the surface of the NPs, thereby increasing the electrical resistance of the sensor. The presence of humidity during the sensing measurements leads to interactions between water molecules and ionosorbed oxygen species, forming terminal hydroxyl groups and releasing electrons back to the sensing material.^45^ When the sensor is exposed to CO_2_, CO_2_ molecules interact with the inosorbed oxygen species or hydroxyl groups on the sensor surface to form carbonate-related species.^46^ These carbonate species modify the surface charge state, leading to changes in the depletion layer width and, consequently, the sensor resistance. A precise determination of the carbonate species formed in our material and their correlation with hydroxyl species would require *operando* studies,^23,25^ which are beyond the scope of this contribution.

To verify the long-term stability and repeatability of the fabricated sensor, we conducted additional sensing measurements on the same electrode without any further treatment in between the experiments. Figure S6 shows the sensor’s behavior during two measurements, each corresponding to three exposure cycles of CO_2_ between 250 and 4000 ppm, labeled as sensor-1 and sensor-1r. As shown in Figure S6a, the sensor signals remain similar for the two consecutive sensing experiments, in particular, for the small concentrations of CO_2_ (<2000 ppm). These results suggest the preservation of the signal over a long period of operation exceeding 140 h. Moreover, no significant changes in the response and recovery time were observed (see Figure S6b,c), indicating high long-term stability and repeatability of the fabricated sensor at RT and 30% RH. Finally, we tested the reproducibility of the sensor fabrication method. In Figure S6a new sensor electrode (sensor-2) was fabricated, which exhibits high reproducibility in CO_2_ sensing performance, particularly in terms of sensor signal and response/recovery times. Table 1 presents the CO_2_ sensing performance of various chemiresistive sensors. The CoFe_2_O_4_ NPs-based sensor exhibits high sensor signal to CO_2_ that is comparable or higher than that of other reported gas sensors, while offering the advantage of operation at RT. These findings indicate the potential of CoFe_2_O_4_ NPs for efficient CO_2_ detection with reduced power consumption, as well as excellent long-term stability and reproducibility. It is worth mentioning that our sensor is sensitive to CO_2_ when operated at 100 °C. However, the concentration-dependent behavior is lost, highlighting its efficiency when operated at room temperature (RT) (see Figure S7).

**Table 1:** Overview of the Sensing Performance of Chemiresistive CO_2_ Sensors

sensor material	operating temperature (°C)	RH (%)	conc. (ppm)	sensor signal^b^	response time (t_90_)	recovery time (*t*_90_)	ref
La_2_O_3_ loaded SnO_2_	400	dry	2030	∼2.5	quick (NR)	NR	^27,28^
CuO/CuFe_2_O_4_	250	dry	5000	1.4	55 min	8 min	^30^
NiFe_2_O_4_ thin film	RT	dry	2000	1.3	100 s	400 s	^33^
CaFe_2_O_4_	350	dry	5000	1.3	70 s	NR^a^	^31^
P[VBTMA][PF_6_]/La_2_O_2_CO_3_	RT	50	2400	1.12^c^	5 min	NR^a^	^15^
ZnO/NiO/Pt	200	14	10,000	3.3%^d^	8.6 min	8.1 min	^47^
50% La-ZnO	400	dry	5000	1.65	90 s	38 s	^48^
Co-doped LaFe_2_O_3_	220	51.9	5000	1.0669	6 s	42 s	^24^
ZnO/CuO	RT	NR^a^	1000	1.097	4.2 min	3.5 min	^49^
SnO_2_	240	14	2000	1.24	350 s	4s	^50^
La_2_O_2_CO_3_	320	dry	2500	1.56	53 s	120 s	^51^
CoFe_2_O_4_ NPs	RT	30	3000	1.18	∼11.25 min	∼12.8 min	this work

a NR: not reported.

b Sensor signal defined as *R*_air_/*R*_CO2_ or *R*_CO2_/*R*_air_.

c Values estimated from the graphs.

d Signal reported as *S* = (*R*_air_ – *R*_CO2_)/*R*_air_ × 100.

We investigated the sensor’s performance in varying humidity, from dry air (0% RH) to 50% RH, as shown in Figures 3 and S8. Figure S8 shows that as RH increases, the baseline resistance decreases from 8.25 GΩ at 0% RH to 1.35 GΩ at 30% RH and 0.59 GΩ at 50% RH, which is a typical behavior of metal oxide-based chemiresistive sensors, along with the dual-sensitivity nature of spinel oxides to both humidity and gas molecules.^52^ Moreover, the sensor exhibits humidity-dependent behavior. At 0 and 30% RH, the sensor’s resistance increases upon CO_2_ exposure, whereas at 50% RH, the sensor exhibits the opposite behavior, with the resistance decreasing upon CO_2_ exposure, as exemplified in Figure 3a,b. This behavior can be attributed to a possible change in the dominant sensing mechanism due to increased humidity levels. When the humidity level is low (up to 30% RH), water molecules adsorb onto the sensing surface by interacting with ionosorbed oxygen species but without fully covering the surface, allowing the sensor to retain a similar mechanism as in dry air, which is primarily based on electronic conduction. In contrast, we hypothesize that increasing the humidity to 50% RH leads to larger coverage of the sensor’s surface with water molecules, forming a hydrogen-bonded network that enables proton transport via the Grotthuss mechanism.^53,54^ This introduces a parallel conduction pathway, leading to a decrease in the resistance upon CO_2_ exposure. This interpretation is consistent with classical studies on spinel-type ceramic humidity sensors,^52^ which show that increasing humidity leads to the formation of adsorbed water layers that facilitate protonic conduction. As a result, the dominant conduction mechanism shifts from electronic-dominated to protonic-dominated transport.

**Figure 3:**
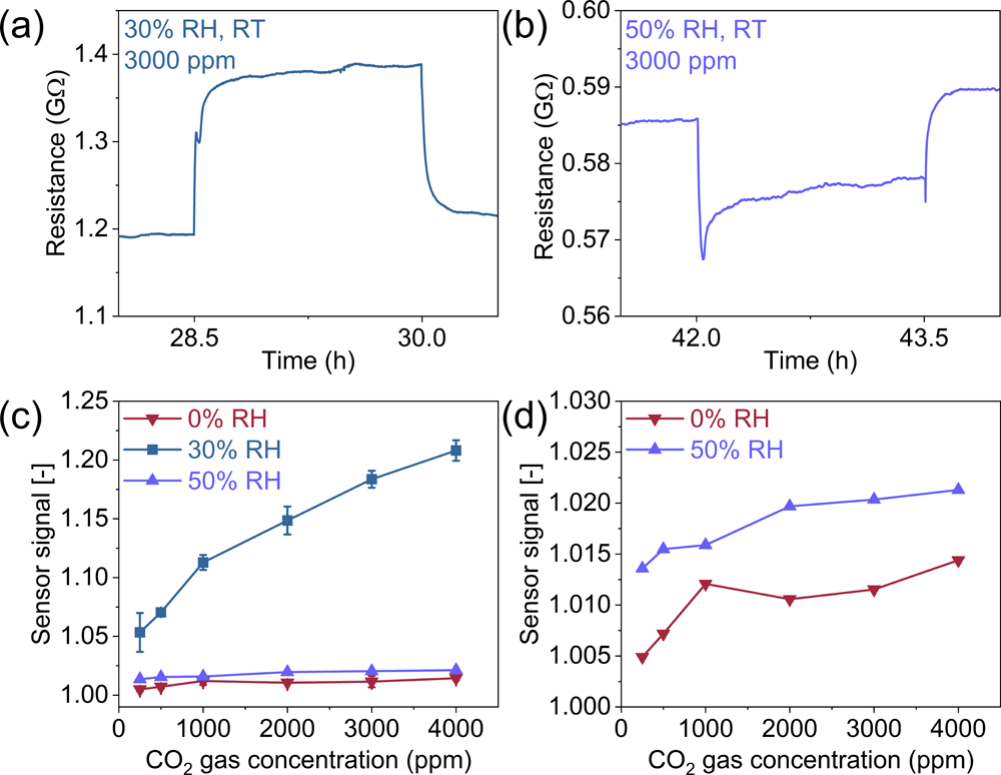
CO_2_ sensing performance of CoFe_2_O_4_ NPs under varying humidity (0, 30, and 50% RH defined at 20 °C) at RT. (a) Resistance profile for 3000 ppm of CO_2_ at 30% RH, showing an increase in resistance upon CO_2_ exposure. (b) Resistance profile for 3000 ppm of CO_2_ at 50% RH, exhibiting a decrease in resistance upon gas exposure. (c) Sensor signal as a function of CO_2_ concentration at different RH levels. (d) Magnified sensor signal at 0 and 50% RH from figure (c).

Figure 3c,d exhibit the CO_2_ sensing performance of the CoFe_2_O_4_-based sensor under different humidity levels. The highest signals are observed during operation at 30% RH, evidencing that water plays a role in the sensing properties as long as it does not fully cover the surface, as it occurs at 50% RH. Under dry (0% RH) and high-humidity (50% RH) conditions, the sensor exhibits markedly lower signals to CO_2_, as seen in Figure 3c. Although the response and recovery times at 0 and 30% RH are comparable, both parameters decrease significantly at 50% RH, suggesting that the change in the sensing mechanism at higher humidity also affects the sensor’s kinetics (Figure S9).

To gain an in-depth understanding of the CO_2_ sensing properties of the CoFe_2_O_4_ NPs, we investigate their N_2_ physisorption isotherm to determine their specific surface area, total pore volume, and pore size distribution. Figure 4a displays the N_2_ adsorption–desorption isotherm of the CoFe_2_O_4_ NPs, which corresponds to a type IVa isotherm. In the relative pressure range of 0.05–0.30 (*p*/*p*_0_) (cf. Figure 4b), the sample exhibits a high BET surface area of 119 m^2^/g. The total pore volume is 0.17 cm^3^/g. In Figure 4c, we observe that the pore size distribution is approximately between 4 and 10 nm, with a dominant peak centered at ca. 5.2 nm. Notably, this pore size distribution closely matches the size distribution of the CoFe_2_O_4_ NPs, indicating that the observed porosity arises mainly from interparticle voids rather than internal pores within the NPs. This suggests that the NPs are nonporous internally, and that the adsorption measurements reflect the textural porosity formed by the packing of nanoparticles. These interparticle voids provide accessible pathways for gas diffusion, which, together with the high surface area for adsorption, are highly beneficial for the gas sensing performance.

**Figure 4:**
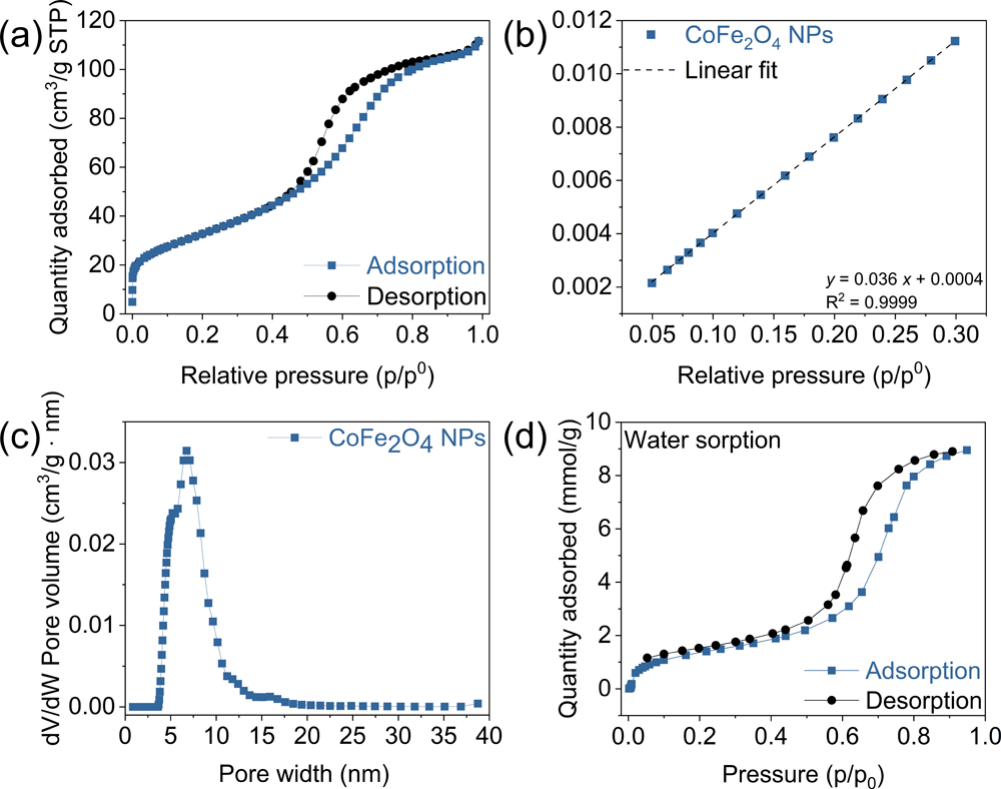
Adsorption and desorption behavior of the CoFe_2_O_4_ NPs. (a) Nitrogen physisorption isotherm. (b) BET plot showing the linear region used to calculate the specific surface area. (c) Corresponding pore size distribution from DFT analysis of the adsorption branch of the N_2_ isotherm. (d) Water vapor sorption isotherm at 25 °C. The water uptake is ∼1.58 mmol/g at *p*/*p*_0_ = 0.3 and ∼2.22 mmol/g at *p*/*p*_0_ = 0.5.

In order to support the proposed humidity-dependent sensing mechanism, we study the adsorption and desorption behavior of CoFe_2_O_4_ NPs toward water vapor. As displayed in Figure 4d, the water sorption isotherm (type IV-like) indicates that at low *p*/*p*_0_ values (<0.3), the water uptake is relatively low (below 1.6 mmol/g), corresponding primarily to surface-bound water. At *p*/*p*_0_ = 0.3 (corresponding to ∼30% RH at the measurement temperature), the adsorption reaches nearly one monolayer. Upon further increasing the relative pressure, a progressive increase in adsorbed water is observed, which is consistent with the formation of multilayer water adsorption.^55,56^ From *p*/*p*_0_ values above 0.5, we observe a pronounced increase in water adsorption accompanied by a hysteresis loop, indicating the onset of capillary condensation.^57,58^ This high-uptake regime occurs at relative pressures comparable to the humidity level (∼50% RH) at which the CO_2_ sensing behavior changes. These results are consistent with the experimental CO_2_ sensing observations, indicating that a change in the water adsorption state occurs when increasing the humidity levels from 30 to 50% RH.

Finally, we investigate the effect of NP size on the CO_2_ sensing performance. In Figure 5, we compare the dynamic resistance curve of our CoFe_2_O_4_ NPs (6 nm) with that of bulk CoFe_2_O_4_ particles (crystallite size ∼ 60 nm) at 30% RH. The resistance curves during exposure to CO_2_ reveal different behavior for the two sensors. While the CoFe_2_O_4_ NPs exhibit high sensitivity across all CO_2_ concentrations (250–4000 ppm), the sensor based on bulk CoFe_2_O_4_ shows no evident signal upon CO_2_ exposure. Furthermore, the baseline resistance of bulk CoFe_2_O_4_ is 17-fold higher than that of the NPs. We propose that the differences in CO_2_ detection arise from the distinct microstructure and surface accessibility of the two sensing layers rather than from a specific intrinsic size threshold. The large, dense and relatively impermeable particles in the bulk CoFe_2_O_4_-based sensor can hinder gas diffusion through the sensing film and limit the formation of reactive oxygen species, reducing the number of active sites. Consequently, the interaction between CO_2_ molecules and the active surface is less effective. On the other hand, the small NPs of 6 nm form a porous sensing layer with a high accessible surface area, which facilitates gas diffusion and enhances the active adsorption sites for both oxygen species and CO_2_ molecules. As a result, the NPs-based sensor exhibits a more efficient CO_2_ sensing performance, including high signals to CO_2_ combined with a lower baseline resistance.

**Figure 5:**
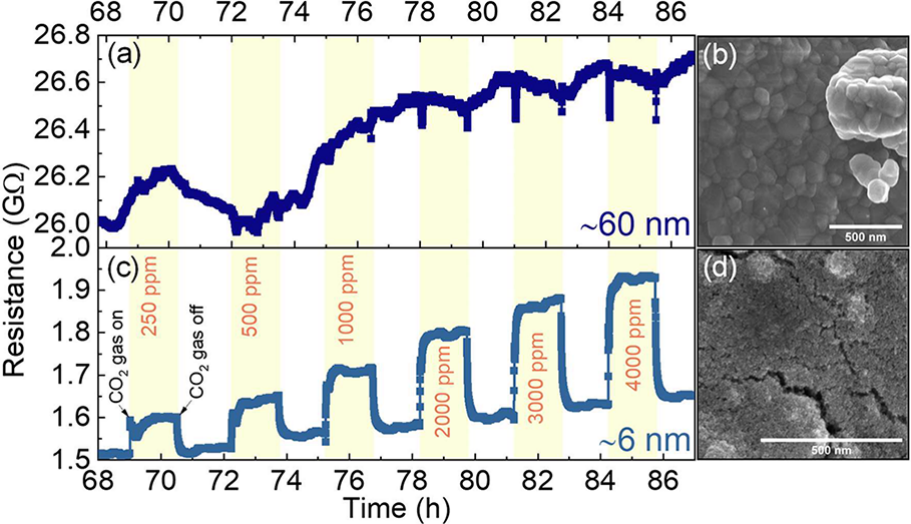
Effect of the particle size on CO_2_ sensing performance at 30% RH (at 20 °C) and RT. (a) Resistance changes upon exposure to CO_2_ (250–4000 ppm) for the sensor based on bulk CoFe_2_O_4_ (crystallite size 60 nm). (b) Corresponding SEM image of the sensing film composed of bulk particles. (c) Resistance variation for the CoFe_2_O_4_ NPs (6 nm). (d) Corresponding SEM image of the NP-based sensing film.

## 4 Conclusions

In summary, we have demonstrated that nanostructured CoFe_2_O_4_ is an efficient room-temperature sensing material for CO_2_ detection across the relevant practical range of 250–4000 ppm. The sensors exhibit high sensor signal, prolonged stability, and excellent reproducibility. Notably, the sensor’s response depends on humidity, with 30% RH providing optimal sensing performance, while increasing the humidity to 50% RH lowers the sensor signal to CO_2_ and alters the sensing mechanism, reflecting the influence of water-assisted surface processes. We therefore propose a change in the sensing mechanism from electronic-dominated to protonic-dominated transport by increasing humidity levels. Furthermore, comparison with bulk CoFe_2_O_4_ (larger crystallite size) highlights the superior sensing performance of small NPs, attributed to the enhanced surface area and porosity, leading to an increased number of active adsorption sites. These findings establish CoFe_2_O_4_ NPs as a promising low-power platform for ambient CO_2_ monitoring and offer valuable insights into the influence of particle size and humidity on chemiresistive gas sensing mechanisms.

## Supplementary Material



## Notes

Additional characterization, including SEM, EDX, PXRD, PDF refinement results, and complementary CO_2_ sensing measurements (PDF)
