# Supplementary material for: Room-Temperature Carbon Dioxide Gas Sensor Based on Co-Ferrite Nanoparticles
Source: ACS Omega. 2026 Feb 18;11(8):13676–83. doi: 10.1021/acsomega.5c11563 (PMC12961461; doi:10.1021/acsomega.5c11563)
Supplement: Supplementary file 1 [file ao5c11563_si_001.pdf]

Supporting Information

Room temperature carbon dioxide gas sensor based on Co-ferrite nanoparticles

Yogesh Mahor,[1] Dorota Koziej,[1,2] Cecilia A. Zito[1,2]*

[1]*Center for Hybrid Nanostructures (CHyN), Institute of Nanostructure and Solid State Physics, University of Hamburg, Luruper Chaussee 149, 22761 Hamburg, Germany*

[2]*The Hamburg Center for Ultrafast Imaging, 22761 Hamburg, Germany*

Corresponding author:

*Cecilia A. Zito; E-mail: cecilia.zito@uni-hamburg.de

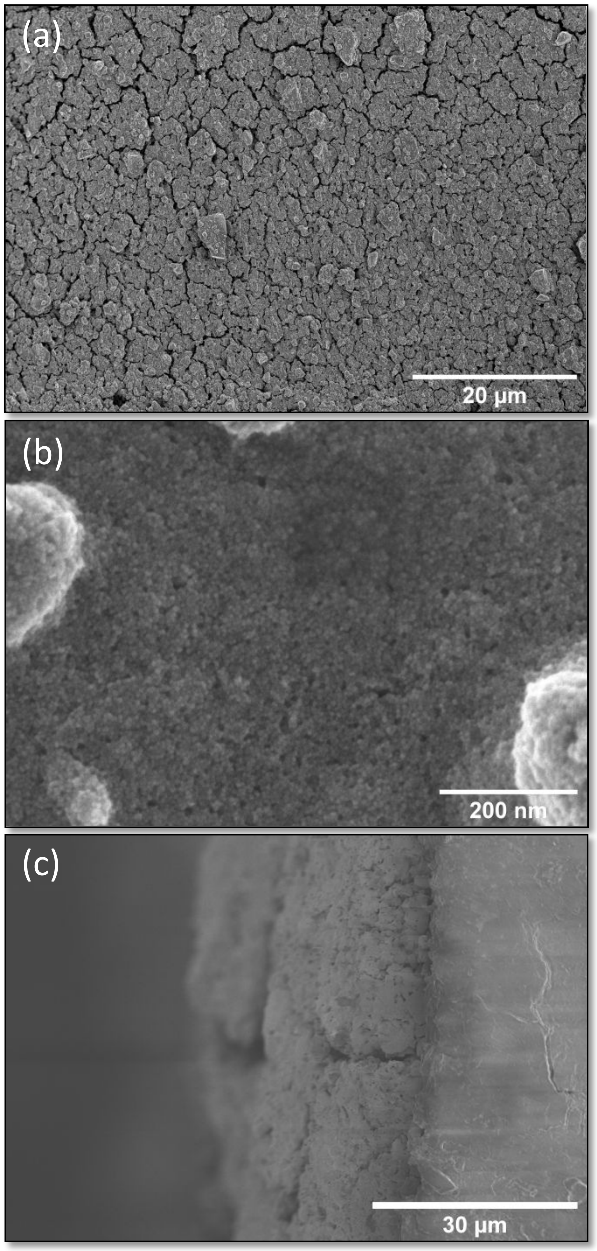

Figure S1: SEM images of the $CoFe_2O_4$ NPs film on Pt-IDE on alumina of the sensor: (a) top view, (b) higher-magnification view, (c) cross-section of film.

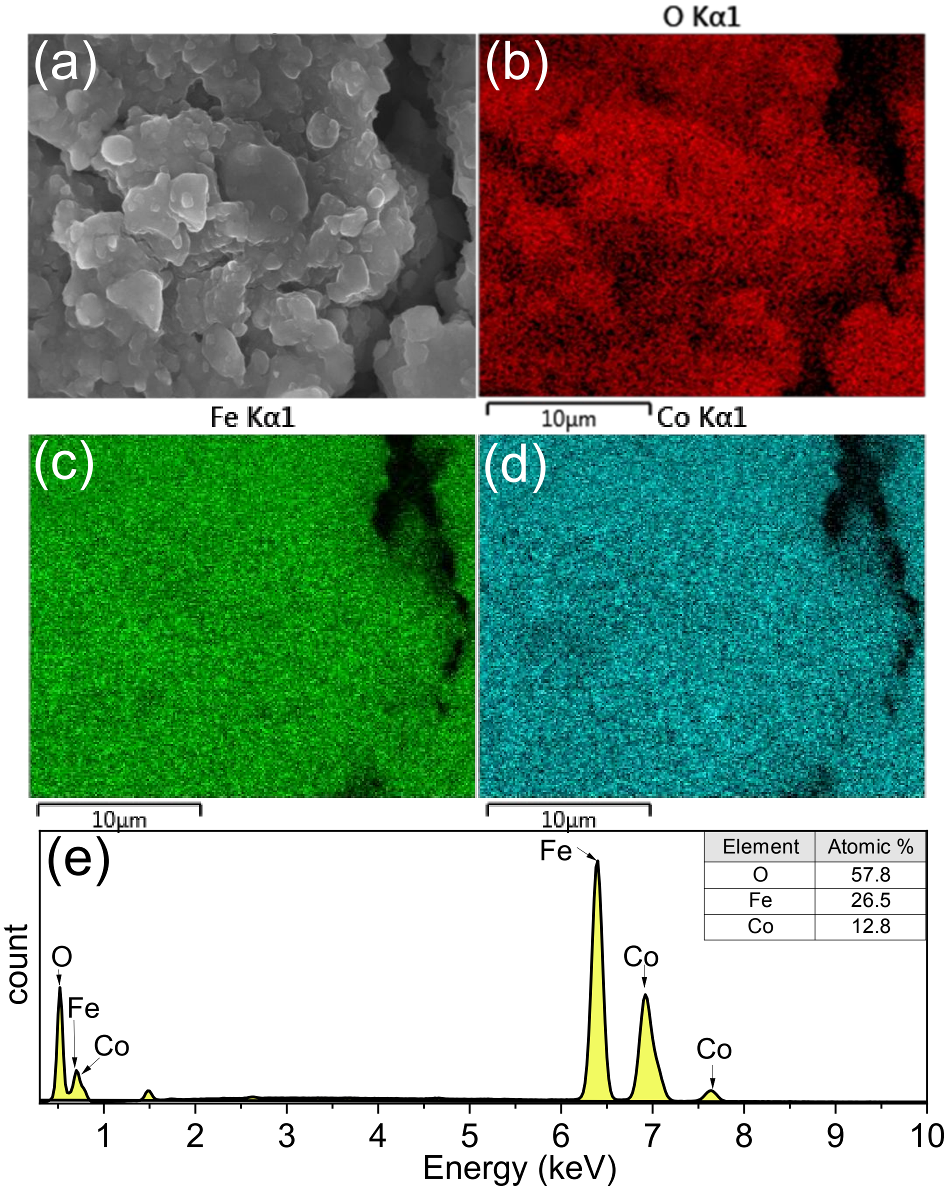

Figure S2: Elemental composition of $CoFe_2O_4$ NPs film on Pt-IDE on alumina. (a) SEM image of the mapped area. EDS elemental mapping of(b) Oxygen $K\alpha_1$, (c) Iron $K\alpha_1$, (d) Cobalt $K\alpha_1$.(e) EDS spectrum, showing the elemental quantification in the inset. The remaining composition consists of 2.6 at% Al, 0.1 at% Si, and 0.1 at% Cl.

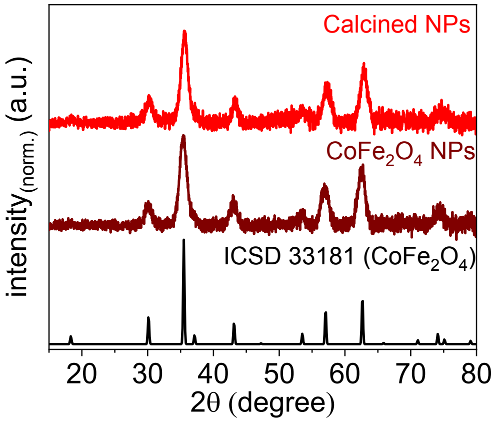

Figure S3: PXRD patterns of as-synthesized $CoFe_2O_4$ NPs and $CoFe_2O_4$ calcined at 400˚C with 5˚C/min heating rate for 1.5 hours.

Table S1: Refined parameters for the PDF data in the r range between 1.5 and 70 Å.

Parameter	Single phase	Two phases	
**a (Å)**	8.39800	8.39802	
**Scale factor**	0.515155	0.403081	0.160522
**Delta2 (Å²)**	2.91022	2.14908	
**U_{iso} Fe/Co (Å²)**	0.00559862	0.00539044	
**U_{iso} O (Å²)**	0.0156495	0.0157597	
**spd (spherical particle diameter) (Å)**	66	76	29
**Fraction (%)**	100	72	28
**Goodness of the fit, Rw**	0.1163	0.1035	

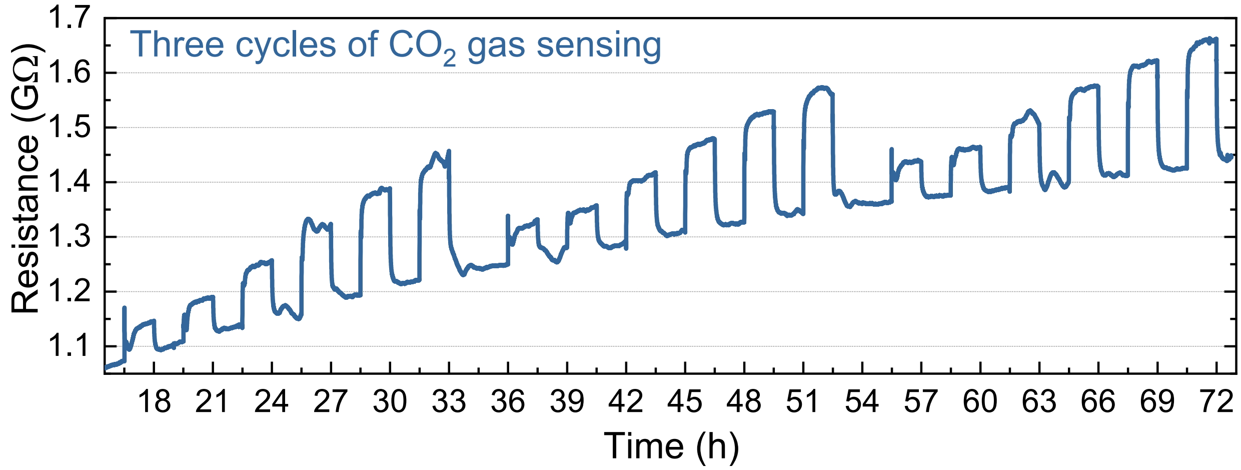

Figure S4: Long-term stability of the sensor. Changes in resistance of the $CoFe_2O_4$ NPs-based sensor over three consecutive CO_2 gas exposure cycles (250-4000 ppm) at RT and 30% RH.

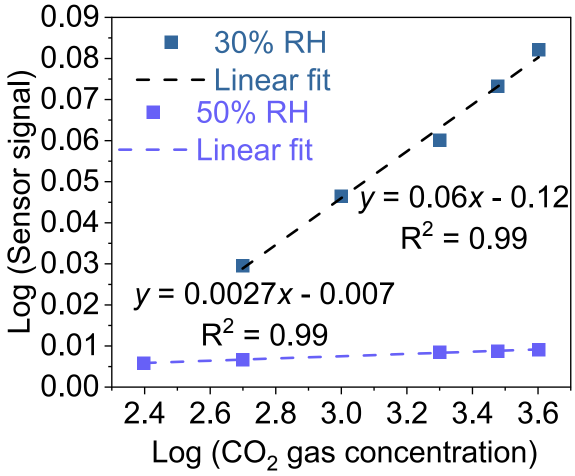

Figure S5: Linear fit of the Log(Sensor Signal) versus Log(Concentration) for $CoFe_2O_4$ NPs-based sensor CO_2 gas exposure cycles (250-4000 ppm) at RT and 30% RH and 50% RH.

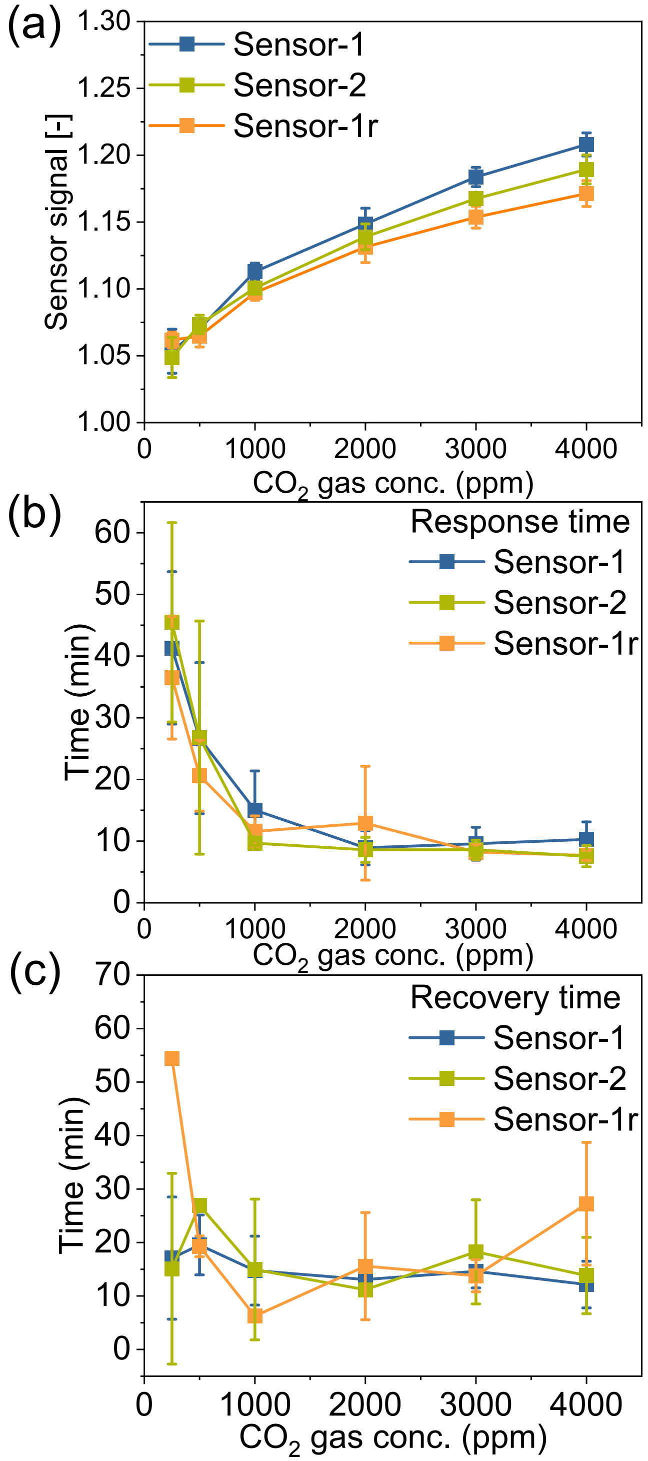

Figure S6: Reproducibility of the sensing performances of the $CoFe_2O_4$ NPs based sensor at 30% RH and RT. While sensor-1 and sensor-1r correspond to the same electrode but tested for two different sensing measurement cycles, sensor-2 corresponds to a new electrode in order to elucidate the reproducibility of the electrode fabrication method. (a) Sensor signal as a function of CO_2 concentration. (b) Response time for each CO_2 concentration, (c) Recovery times of the three sensors as a function of CO_2 concentration

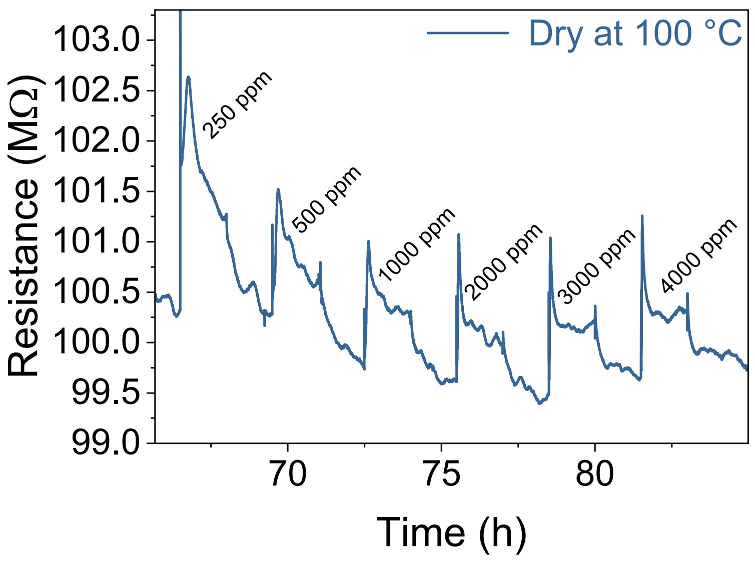

Figure S7: CO_2 sensing performance of $CoFe_2O_4$ NPs in dry air at 100°C. By increasing the operating temperature to 100 °C, the sensor based on $CoFe_2O_4$ no longer displays the concentration-dependent behavior towards CO_2.

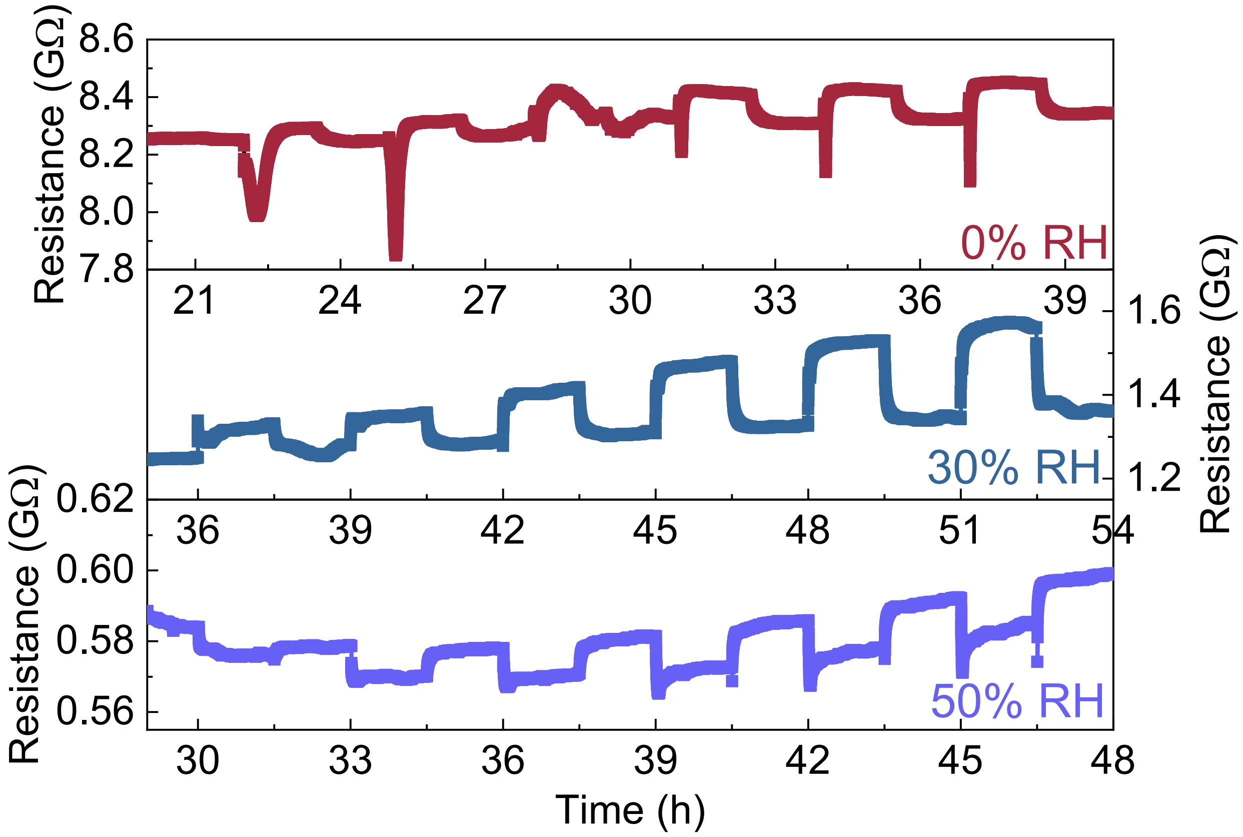

Figure S8: **Effect of humidity levels on the CO_2 sensing performance.** Changes in the resistance of $CoFe_2O_4$ NPs in dry air, 30% RH, and 50% RH at RT for the concentration range of CO_2 between 250 and 4000 ppm.

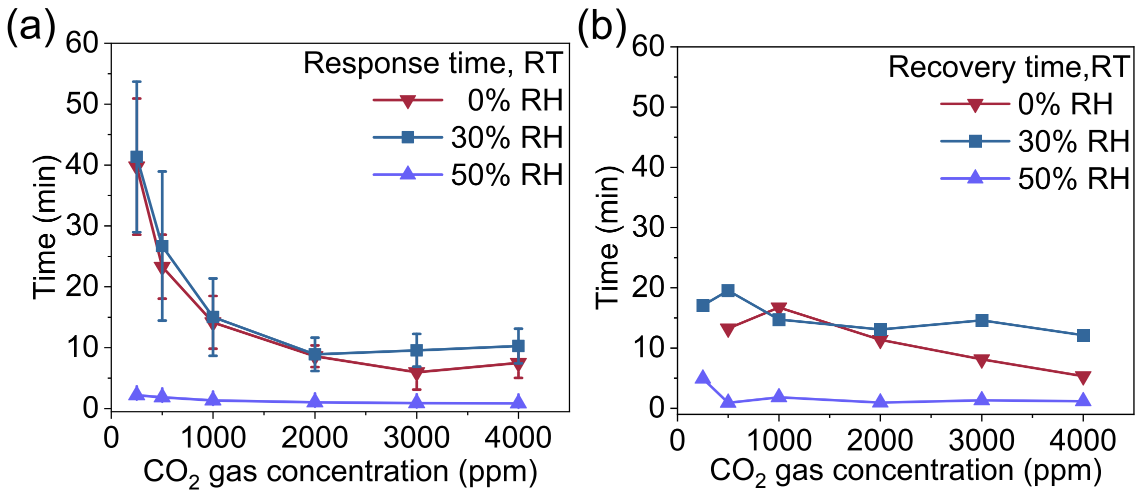

Figure S9: CO_2 sensing performance of $CoFe_2O_4$ NPs in 0% RH, 30% RH and 50% RH at RT as a function of CO_2 concentration: (a) response times, and (b) recovery times.
